# Supplementary material for: Discovery and characterization of variance QTLs in human induced pluripotent stem cells
Source: PLoS Genet. 2019 Apr 19;15(4):e1008045. doi: 10.1371/journal.pgen.1008045 (PMC6474585; doi:10.1371/journal.pgen.1008045)
Supplement: S1 Text — (PDF) [file pgen.1008045.s001.pdf]

Text S1: Derivation of ZINB model

1 Negative binomial model

We assume the count data observed in single cell RNA sequencing are generated by a zero-inflated negative binomial (ZINB) distribution. First, we derive the negative binomial (NB) distribution. Let

- r_{ijk} be the number of molecules for individual i, cell j, gene k
- R_{ij} be a size factor for each cell
- μ_{ik} be proportional to relative abundance
- ϕ_{ik} be the variance of expression noise

Then, under the negative binomial model

$$r_{ijk} \sim \text{Poisson}(\cdot; R_{ij}\lambda_{ijk}) \tag{1}$$
$$\lambda_{ijk} \sim \text{Gamma}(\cdot; \mu_{ik}, \phi_{ik}) \tag{2}$$

where $\text{Gamma}(\cdot; \mu, \phi)$ denotes the Gamma distribution with mean μ, variance $\mu^2\phi$, and density

$$\text{Gamma}(x; \mu, \phi) = \frac{(\phi^{-1}\mu^{-1})^{\phi^{-1}}}{\Gamma(\phi^{-1})} x^{\phi^{-1}-1} \exp(\phi^{-1}\mu^{-1}x) \tag{3}$$

This parameterization is derived by assuming that each cell has a latent expression value λ_{ijk}, and the observed molecule counts are generated by a Poisson technical noise model describing the sequencing experiment [17].

We further assume that the collection of true expression values λ_{ijk} for each individual i and gene k are described by a common mean μ_{ik} and multiplicative Gamma distributed noise with mean 1 and variance ϕ_{ik}. Using standard results of the Gamma distribution, the resulting distribution over λ_{ijk} is also Gamma distributed.

Marginalizing over the latent expression λ_{ijk}, the log likelihood of each observation is:

$$l_{\mathrm{NB}}(r_{ijk}) = \ln p(r_{ijk} \mid \cdot) = r_{ijk} \ln \left(\frac{R_{ij}\mu_{ik}}{R_{ij}\mu_{ik} + \phi_{ik}^{-1}} \right) + \phi_{ik}^{-1} \ln \left(\frac{\phi_{ik}^{-1}}{R_{ij}\mu_{ik} + \phi_{ik}^{-1}} \right) + \ln \Gamma(r_{ijk} + \phi_{ik}^{-1}) - \ln \Gamma(r_{ijk} + 1) - \ln \Gamma(\phi_{ik}^{-1}) \quad (4)$$

2 Zero-inflated negative binomial model

Under the ZINB model

$$r_{ijk} \sim \mathrm{Poisson}(\cdot; R_{ij}\lambda_{ijk}) \quad (5)$$

$$\lambda_{ijk} \sim \pi_{ik}\delta_0(\cdot) + (1 - \pi_{ik})\mathrm{Gamma}(\cdot; \mu_{ik}, \phi_{ik}) \quad (6)$$

where π_{ik} denotes the proportion of excess zeros.

This model is derived by simply assuming that the distribution of latent gene expression values is point-Gamma distributed rather than Gamma distributed.

Considering just the non-zero component of (6), marginalizing out λ yields (4), adjusted by the mixture weight:

$$l(\cdot) = \ln(1 - \pi_{ik}) + l_{\mathrm{NB}}(\cdot) \quad (7)$$

Considering the zero component, we note that the Poisson distribution with rate parameter zero is a point mass on zero. Therefore, the likelihood of observations not equal to zero under this component is zero, and the likelihood of observations equal to zero is π_{ik}.

Therefore, marginalizing over the point-Gamma mixture yields the log likelihood:

$$l_{\mathrm{ZINB}}(\cdot) = \ln(\pi_{ik} + \exp(l(\cdot))) \text{ if } r_{ijk} = 0 \quad (8)$$

$$l_{\mathrm{ZINB}}(\cdot) = l(\cdot) \text{ otherwise} \quad (9)$$

where $l(\cdot)$ is defined as in (7).

3 Technical confounding model

To account for confounding, we change the technical noise model. Let

- $\mathbf{x}_{ij}$ be a q-vector of confounders per cell
- $\boldsymbol{\beta}_k$ be a q-vector of confounding effects on gene k

Then, we assume

$$r_{ijk} \sim \text{Poisson}(\cdot; R_{ij} \exp(\mathbf{x}'_{ij}\boldsymbol{\beta}_k)\lambda_{ijk}) \tag{10}$$

The fundamental assumption underlying this technical noise model is that each cell has some true, latent expression value λ_{ijk}. The confounding process described by $\exp(\mathbf{x}'_{ij}\boldsymbol{\beta}_k)$ corresponds to the effect of cell preparation, library preparation, etc. on the gene expression value which can be observed prior to the sequencing step, and Poisson sampling corresponds to performing the sequencing step. We do not model technical confounding on the mean μ_{ik}, the dispersion ϕ_{ik}, or the proportion of zeros π_{ik} because these parameters describe the true, latent gene expression values λ_{ijk}, which are unchanged by technical confounding.

Under this assumption, we derive the log likelihood of the data under (10) by simply multiplying $R_{ijk}\mu_{ik}$ by $\exp(\mathbf{x}'_{ij}\boldsymbol{\beta}_k)$ throughout (4).

4 Estimating expression mean and variance

We can derive the mean and variance of latent gene expression values λ_{ijk} by introducing latent indicator variables z_{ijk} which denote which component of the point-Gamma mixture each observation came from.

Then

$$\begin{aligned}
\mathbb{E}[\lambda_{ijk}] &= \mathbb{E}[\mathbb{E}[\lambda_{ijk} \mid z_{ijk}]] && (11)\\
&= \pi_{ik}(0) + (1 - \pi_{ik})\mu_{ik} && (12)\\
\mathbb{E}[\lambda_{ijk}] &= (1 - \pi_{ik})\mu_{ik} && (13)
\end{aligned}$$

and

$$\begin{aligned}
\mathbb{V}[\lambda_{ijk}] &= \mathbb{E}[\mathbb{V}[\lambda_{ijk} \mid z_{ijk}]] + \mathbb{V}[\mathbb{E}[\lambda_{ijk} \mid z_{ijk}]] && (14)\\
\mathbb{E}[\mathbb{V}[\lambda_{ijk} \mid z_{ijk}]] &= \pi_{ik}(0) + (1 - \pi_{ik})\mu_{ik}^2\phi_{ik} && (15)\\
\mathbb{V}[\mathbb{E}[\lambda_{ijk} \mid z_{ijk}]] &= \mathbb{E}_z[(\mathbb{E}[\lambda_{ijk} \mid z_{ijk}] - \mathbb{E}_z[\lambda_{ijk}])^2] && (16)\\
&= \pi_{ik}[(1 - \pi_{ik})\mu_{ik}]^2 + (1 - \pi_{ik})[\pi_{ik}\mu_{ik}]^2 && (17)\\
&= \pi_{ik}(1 - \pi_{ik})\mu_{ik}^2 && (18)\\
\mathbb{V}[\lambda_{ijk}] &= (1 - \pi_{ik})\mu_{ik}^2\phi_{ik} + \pi_{ik}(1 - \pi_{ik})\mu_{ik}^2 && (19)
\end{aligned}$$

5 Pitfalls of naive analysis methods

The approach we have developed here should be compared to naive approaches which analyze either counts or log-transformed counts. We argue these naive approaches are

fundamentally flawed as methods of finding variance QTLs. The essence of the flaw is that the mean-variance relationship in the Poisson observation process will induce false positive variance QTLs in the presence of mean QTLs.

To illustrate how false positives arise, consider the (simplistic) case of a gene with zero variance among cells from each individual, and zero variance within each genotype class of some *cis*-variant. In this case, there is no variance in true expression at all, and therefore no variance QTL.

Suppose now the *cis*-variant has a mean effect on that gene, and that the cells in individuals with genotype i have true expression μ_i $(i = 0, 1, 2)$ that are not identical. If we generate sequencing libraries of size R for each cell, then the *observed counts* for an individual with genotype i will be Poisson distributed with mean $R\mu_i$ and therefore variance $R\mu_i$.

Because the μ_i vary across genotype classes, the variances of the observed counts will also vary across genotype classes. But the true variance of gene expression was zero in each individual. Therefore, direct analysis of the counts would yield a false positive vQTL in this setting.

Analyzing log-transformed counts would not solve this problem because there is still a mean-variance relationship in the log of a Poisson distribution. Indeed, let r denote the observed count from a single cell RNA sequencing experiment, and suppose $\mathbb{E}[r] = \mu$ and $\mathbb{V}[r] = \sigma^2$. Then, via second-order Taylor expansion:

$$\mathbb{V}[\ln(r + \epsilon)] \approx \frac{2\sigma^2}{(\mu + \epsilon)^2} - \frac{\sigma^4}{(\mu + \epsilon)^4} \tag{20}$$

These arguments demonstrate the need for deconvolution methods to estimate the distribution of underlying gene expression [17], and map its variance rather than the variance of either the counts or log-transformed counts.
