# Supplementary figures and images for: Discovery and characterization of variance QTLs in human induced pluripotent stem cells
Source: PLoS Genet. 2019 Apr 19;15(4):e1008045. doi: 10.1371/journal.pgen.1008045 (PMC6474585; doi:10.1371/journal.pgen.1008045)

Number of cells by individual

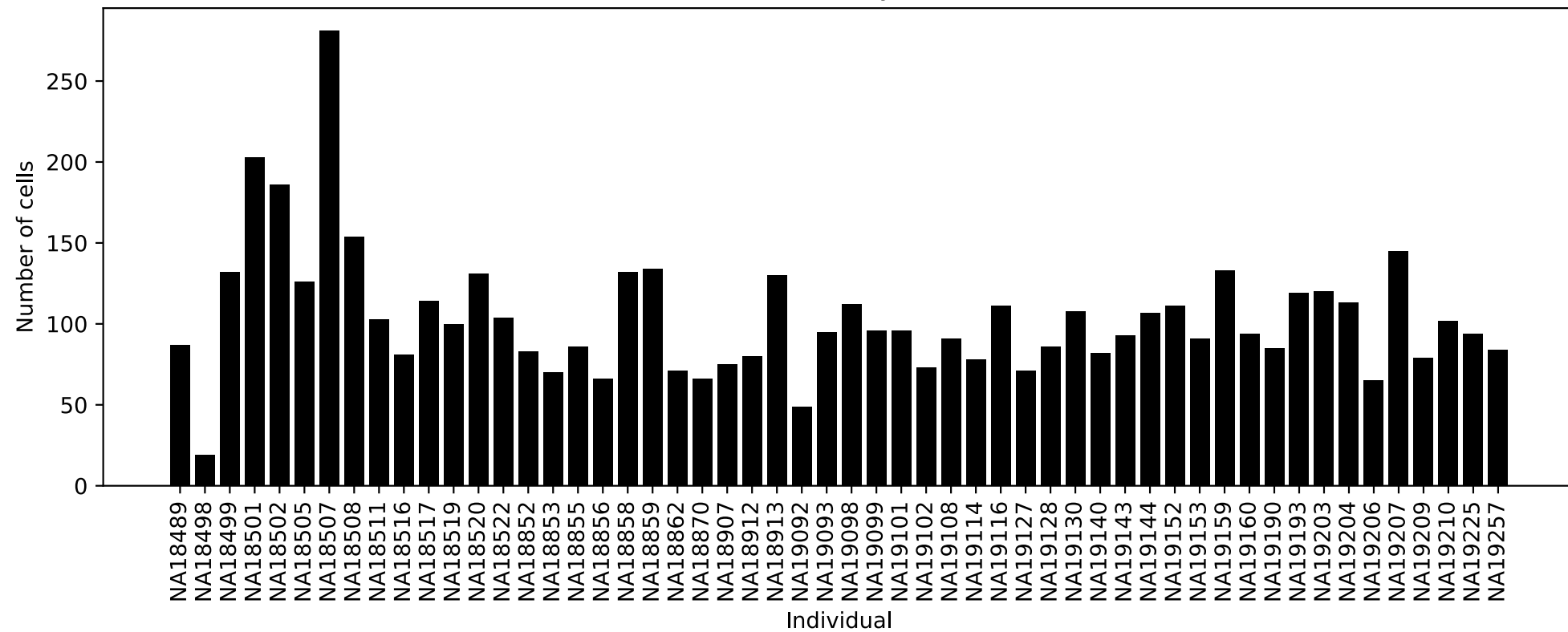

Molecule count by individual

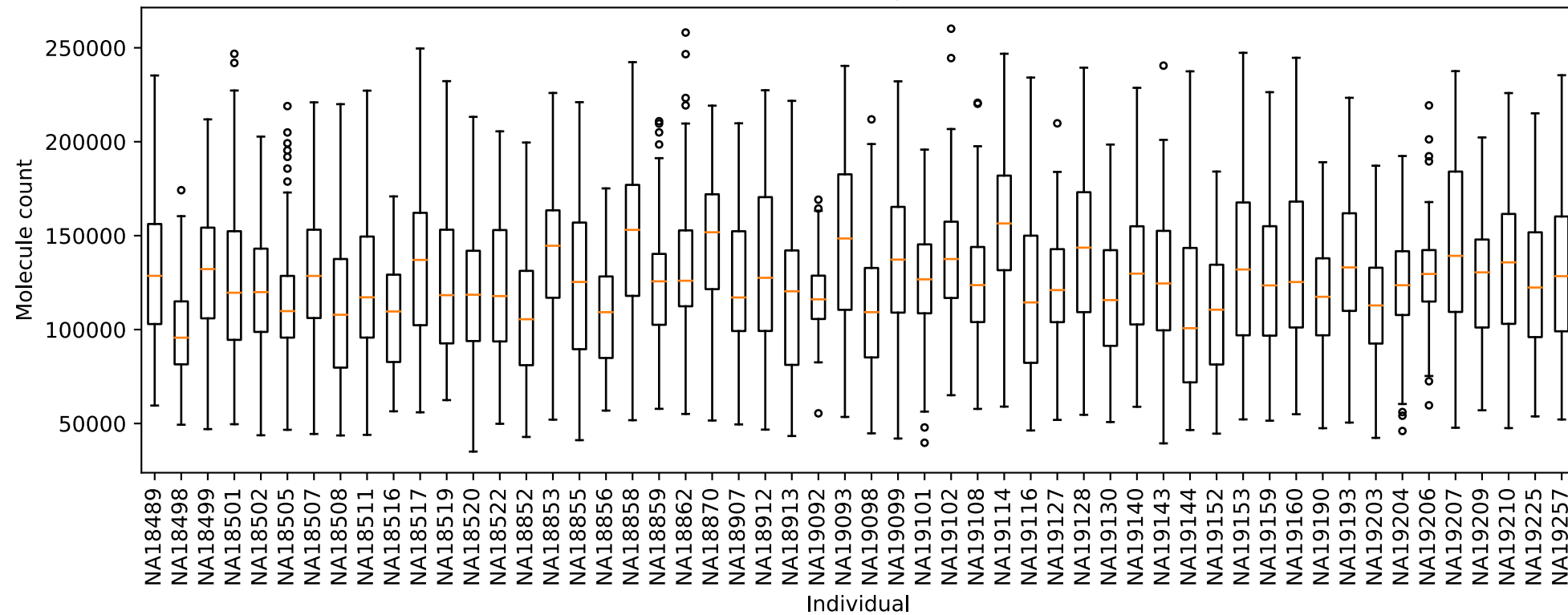

Supplement: S1 Fig — Number of cells per individual, and number of molecules per cell after applying quality control filters. (PDF) [file pgen.1008045.s002.pdf]

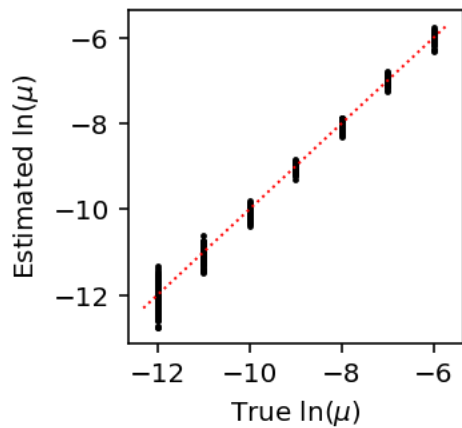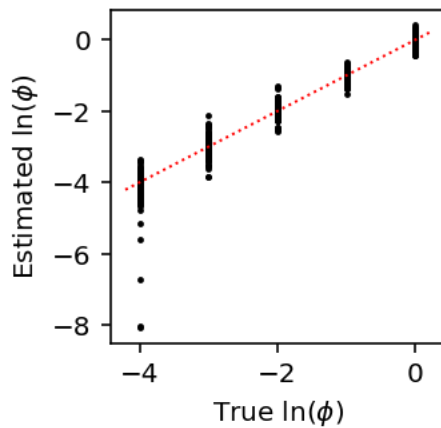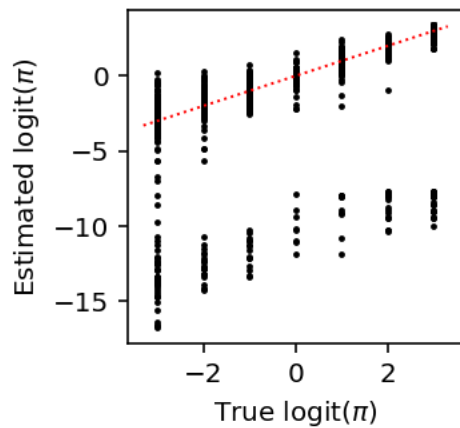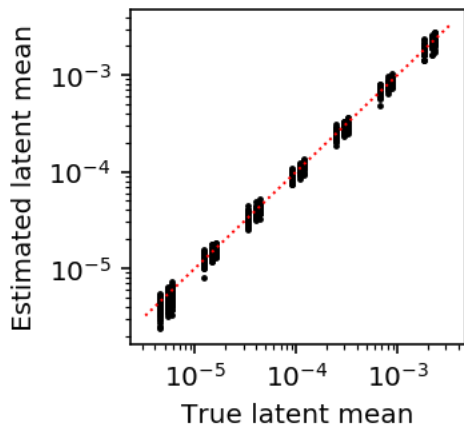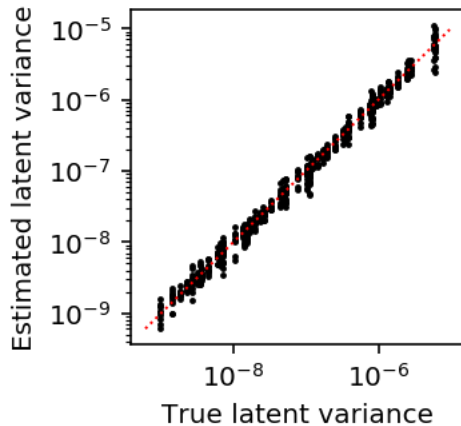

Supplement: S2 Fig — Estimates of ln(μ) and latent mean are displayed for logit(π) < 0. Estimates of ln(ϕ) and latent variance are displayed for ln(μ) > −10, logit(π) < 0. Estimates of logit(π) are displayed over the entire range of parameter values. In each trial, simulated molecule counts for 95 cells are drawn from the model assuming 114,026 molecules per cell, matching the median number of cells, and molecules per cell in the observed data. (PDF) [file pgen.1008045.s003.pdf]

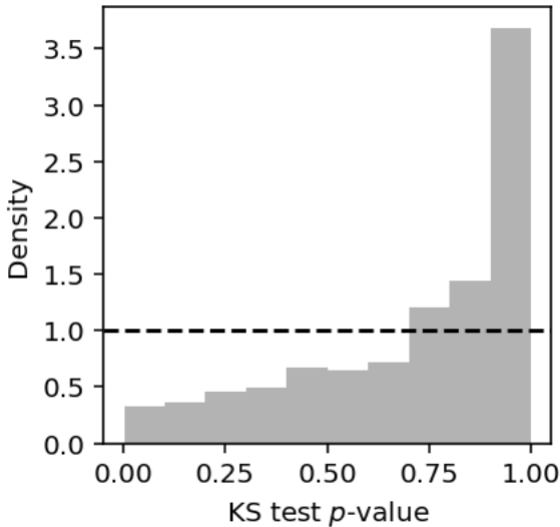

Supplement: S3 Fig — For each simulated data set, we use Kolmogorov-Smirnov test to test for departure of randomized quantiles of the data (based on the fitted ZINB distribution) from the uniform distribution. (PDF) [file pgen.1008045.s004.pdf]

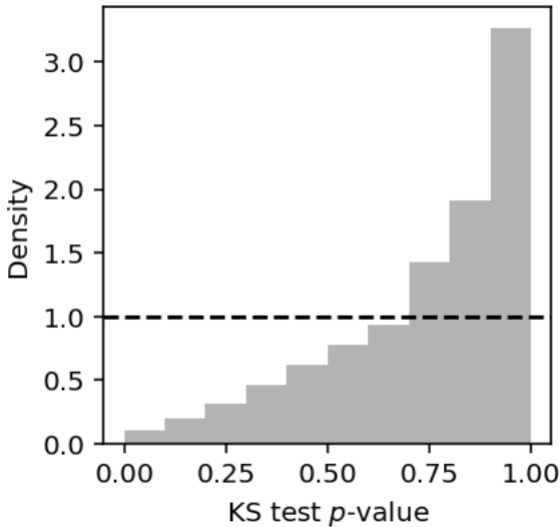

Supplement: S4 Fig — For the set of observed UMI counts for each individual, for each gene, we use Kolmogorov-Smirnov test to test for departure of randomized quantiles of the data (based on the fitted ZINB distribution) from the uniform distribution. (PDF) [file pgen.1008045.s005.pdf]

**Dispersion**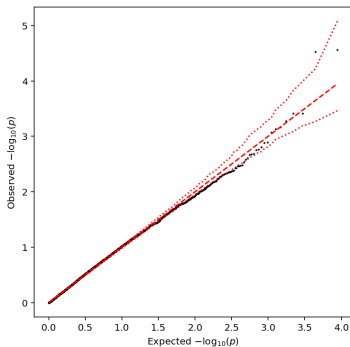**Mean**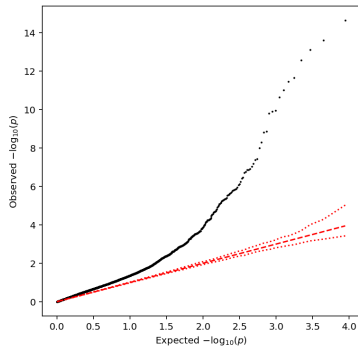**Variance**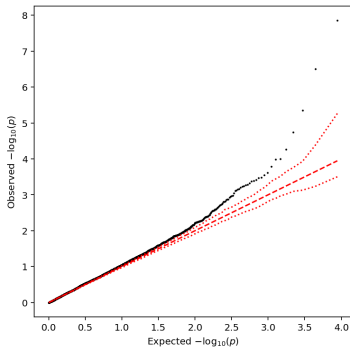**Coefficient of variation**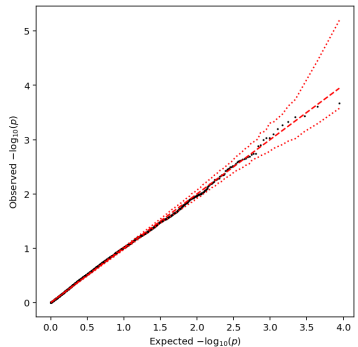**Fano factor**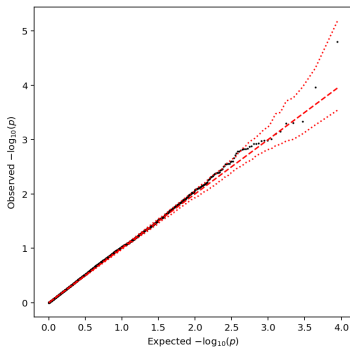**Residualized variance**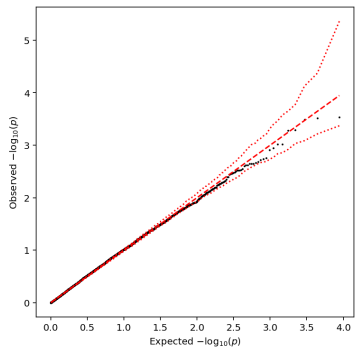

Supplement: S5 Fig — QQ plots are shown for dispersion, mean, variance, coefficient of variation (CV), and Fano factor. (PDF) [file pgen.1008045.s006.pdf]

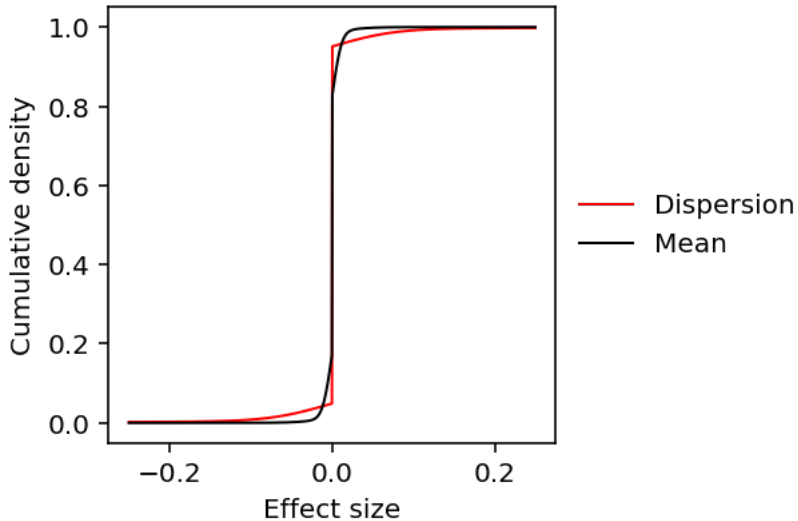

Supplement: S6 Fig — We fit a unimodal mixture of Gaussians to the distribution of observed eQTL (QTL) effect sizes (in terms of log fold change) using Empirical Bayes. (PDF) [file pgen.1008045.s007.pdf]

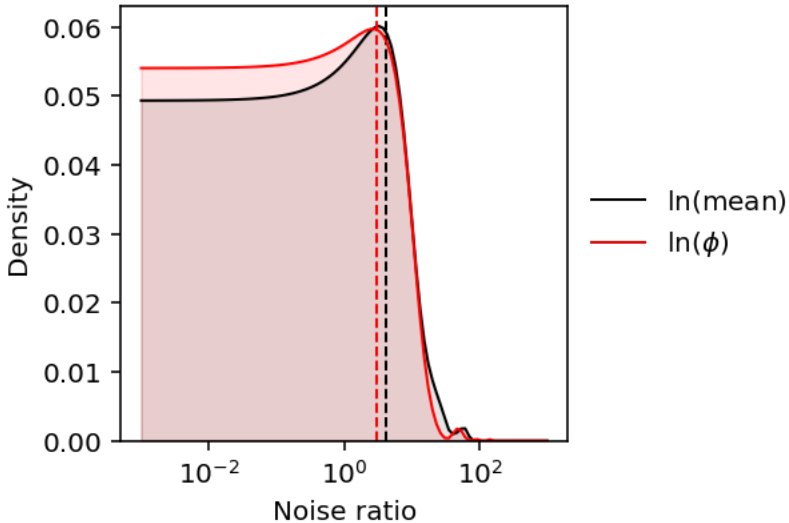

Supplement: S7 Fig — Noise ratios (ratio of measurement error variance to phenotypic variance) are estimated for 200 randomly chosen genes using a two-step empirical Bayes procedure. (PDF) [file pgen.1008045.s008.pdf]
